# Adherence to antiretroviral treatment regimens and correlation with risk of hospitalization among commercially insured HIV patients in the US

**DOI:** 10.1186/1758-2652-13-S4-O3

**Published:** 2010-11-08

**Authors:** PE Sax, JL Meyers, MJ Mugavero, KL Davis

**Affiliations:** 1 Brigham and Women's Hospital, Boston, USA; 2 RTI Health Solutions, Health Economics, Research Triangle Park, USA; 3 University of Alabama at Birmingham, Birmingham, USA

## Purpose

A lower daily pill burden may improve adherence to antiretroviral treatment (ART) and improve outcomes. The goal of this study was to assess differences in adherence rates based on the number of pills taken per day and to evaluate how adherence correlates with the risk of hospitalization.

## Methods

This analysis examined commercially insured patients in the LifeLink claims database. Patients were selected if they had a diagnosis of HIV/AIDS (ICD-9-CM code 042.xx) between 6/1/2006 and 12/31/2008 and received a complete ART regimen defined as 2 nucleoside/nucleotide reverse transcriptase inhibitors (NRTIs) along with a third agent (NNRTI, protease inhibitor, CCR5 antagonist, or integrase inhibitor). Patients were grouped according to their daily pill count: one, two, or three or more pills a day, and were required to receive their regimen for at least 60 days. Outcomes included adherence (both absolute and at pre-specified thresholds) and rates of hospitalization. Adherence was measured as a proportion (medication possession ratio) by dividing the days the patient received a complete regimen by the days between the start and end of the regimen. Logistic regressions were undertaken to assess the relationship between pills per day and adherence and hospitalization while controlling for demographics, comorbidities, and ART-naïve (vs. experienced) status.

## Results

7,073 patients met the study inclusion criteria, among whom 33.4%, 5.8%, and 60.8% received one, two, or three or more pills a day, respectively. 1,829 (20.5%) patients were excluded because they had an incomplete regimen. Approximately 47% of patients receiving one pill a day achieved ≥95% adherence, compared to 41% of patients receiving two pills a day, and 34% of patients receiving three or more pills a day. Based on regression results, patients receiving one pill a day were 61% more likely to reach a 95% adherence threshold vs. patients receiving three or more pills a day (odds ratio [OR]=1.61, P<0.001). Regardless of number of pills per day received, patients were 40% less likely to have a hospitalization if they were adherent to therapy (OR=0.62, P<0.001). Patients receiving one pill a day were 21% less likely to have a hospitalization vs. patients receiving three or more pills a day (OR=0.770, P<0.01).

## Conclusions

We found receiving an ART regimen consisting of one pill a day was associated with better adherence and a lower risk of hospitalization.

